# A Natural Triterpene Derivative from *Euphorbia kansui* Inhibits Cell Proliferation and Induces Apoptosis against Rat Intestinal Epithelioid Cell Line *in Vitro*

**DOI:** 10.3390/ijms160818956

**Published:** 2015-08-12

**Authors:** Fangfang Cheng, Yanjing Yang, Li Zhang, Yudan Cao, Weifeng Yao, Yuping Tang, Anwei Ding

**Affiliations:** 1 Jiangsu Collaborative Innovation Center of Chinese Medicinal Resources Industrialization, and National and Local Collaborative Engineering Center of Chinese Medicinal Resources Industrialization and Formulae Innovative Medicine, Nanjing University of Chinese Medicine, Nanjing 210023, China; E-Mails: cff19870524@163.com (F.C.); raindc@163.com (Y.C.); njweifengyao@163.com (W.Y.); awding105@163.com (A.D.); 2 Medicament Department, Zhangjiagang Hospital of Traditional Chinese Medicine, Suzhou 215600, China; E-Mail: candy00jing@126.com

**Keywords:** kansenone, cell proliferation, cell damage, cell cycle, cell apoptosis, death receptor pathway, mitochondrial pathway

## Abstract

Kansenone is a triterpene from the root of the traditional Chinese medicine, *Euphorbia kansui*. However, kansenone exerts serious toxicity, but the exact mechanism was not clear. In this work, the effects of kansenone on cell proliferation, cell cycle, cell damage, and cell apoptosis were investigated. The suppression of cell proliferation was assessed via the colorimetric MTT assay, and cell morphology was visualized via inverted microscopy after IEC-6 cells were incubated with different concentrations of kansenone. Reactive oxygen species (ROS), superoxide dismutase (SOD) and malondialdehyde (MDA) content were detected for evaluating cell damage. RNase/propidium iodide (PI) labeling for evaluation of cell cycle distribution was performed by flow cytometry analysis. Annexin V-fluorescein isothiocyanate (FITC)/PI and Hoechst 33342/Annexin V-FITC/PI staining assay for cell apoptosis detection were performed using confocal laser scanning microscopy and high content screening. Moreover, apoptosis induction was further confirmed by transmission electron microscope (TEM) and JC-1 mitochondrial membrane potential, western blot and RT-PCR analysis. The results demonstrated that kansenone exerted high cytotoxicity, induced cell arrest at G0/G1 phase, and caused mitochondria damage. In addition, kansenone could up-regulate the apoptotic proteins Bax, AIF, Apaf-1, cytochrome *c*, caspase-3, caspase-9, caspase-8, FasR, FasL, NF-κB, and TNFR1 mRNA expression levels, and down-regulate the anti-apoptotic Bcl-2 family proteins, revealing that kansenone induces apoptosis through both the death receptor and mitochondrial pathways.

## 1. Introduction

The dried root of *kansui* T.N. Liou ex T.P. Wang (called *kansui*) is an effective and commonly used herbal medicine for the treatment of edema, ascites, and asthma [1]. Recently, it was found that *kansui* has pharmacological activities including tumor inhibition [2], anti-viral effects [3,4], immune system regulation [5], modulatory effects on IFN-γ [6], and as a diabetes treatment [7]. Unfortunately, *kansui* has highly toxic side effects in the liver and kidney, which restricts its clinical application [8,9].

Previous studies reported that *kansui* mainly contains diterpenoids [10], triterpenes [11,12], and phenolic derivatives [13]. Diterpenoids were considered to be the major bioactive components of *kansui*, exhibiting antiviral, anticancer and pesticidal effects [14,15]. Song and Duan studied the biochemical responses and hepatocyte cytotoxicity of vinegar-processed *kansui* (VP-*kansui*), indicating that VP-*kansui* caused low perturbations in endogenous metabolism and hepatotoxicity because of decreased contents of diterpene and triterpene [1,9]. However, all these results were based on the cytotoxicity detection of ethanol (EtOH) extract or ethyl acetate (EtOAc) extract, not pure compounds. Ding and co-workers successfully developed a bio-guided isolation method to separate 12 terpernoids from ethyl acetate (EtOAc) extract and simply investigated their gastrointestinal and hepatic cytotoxicity against LO2 and GES-1 cell lines via MTT assay [16]. The results demonstrated kansenone, as a member of the triterpenes, also exhibited strong inhibition of cell proliferation against two human normal cell lines with low IC_50_ values of 14.36 and 13.44 μM, respectively, but the exact mechanism was not clearly represent.

Many physiological growth control mechanisms that regulate cell proliferation and tissue homeostasis are attributed to programmed cell death (apoptosis) processes that usually evoke cell death through intrinsic (via mitochondrial) or extrinsic (via death receptors) pathways [17]. Mitochondria-related apoptosis transports death signals via Bcl-2 family proteins, to trigger depletion of outer membrane potential, release of proteins residing in mitochondrial intermembrane space (MIS) and activation of the caspase family [18]. The activated caspase members included caspase-3, and caspase-9 *etc*. The death receptor-related apoptosis is conducting cell lesion via the death-domain-containing receptors including tumor necrosis factor receptor (TNFR), Fas receptor (FasR), death receptor 3 (DR3, also called Apo3, WSL-1, TRAMP or LARD), death receptor 4 (DR4), death receptor 5 (DR5, also called TRAIL-R2, TRICK2, or KILLER), death receptor 6 (DR6) and their corresponding ligands including tumor necrosis factor (TNF), Fas ligand (FasL), and tumor necrosis factor (TNF)-related apoptosis-inducing ligand (TRAIL) *etc.* [19,20,21]. Cascante and his group have even examined the response of HT29 and Caco-2 colon-cancer cell lines to a new natural triterpene, maslinic acid. They found maslinic acid exerted a significant anti-proliferation effect to HT29 and Caco-2 by inducing an apoptotic process via caspase-3 activation through a p53-independent mechanism, but did not alter the cell cycle or induce apoptosis in the non-tumoural intestine cell lines IEC-6 and IEC-18 [22].

Herein, to examine the cytotoxicity of kansenone to normal tissue, rat intestinal crypt epithelial cell line (IEC-6) was selected as a model cell and the cytotoxicity mechanism of kansenone on IEC-6 was preliminarily investigated. The relative cell viability of kansenone on IEC-6 cells was determined by MTT assay and cell morphology was observed under the inverted phase contrast microscopy, revealing that kansenone had a strong cytotoxicity against intestinal epithelial cells. The results of ROS, SOD activity, and MDA kit showed that kansenone has oxidative damage to IEC-6 via ROS-induced mechanism. Cell cycle and apoptosis of IEC-6 cells treated with kansenone were determined by flow cytometry and confocal laser scanning microscopy, showing that kansenone could arrest IEC-6 cells in G0/G1 phase and induce apoptosis of IEC-6 cells in a concentration-dependent manner. In addition, kansenone caused mitochondrial ultrastructure of IEC-6 cells damaged and mitochondrial membrane potential decreased. Furthermore, kansenone-induced apoptosis is likely to be mediated through the death receptor and mitochondrial pathways, as evidenced by up-regulation of Bax, apoptosis-inducing factor (AIF), the adaptor molecule apoptotic protease activating factor 1 (Apaf-1), and cytochrome *c*, caspase-3, caspase-9, caspase-8 activity, FasR, FasL, NF-κB, and TNF receptor-1 (TNFR1) mRNA expression level and down-regulation of Bcl-2 protein.

## 2. Results and Discussion

### 2.1. Effects of Kansenone on Cell Proliferation and Cell Morphology

**Figure 1:**
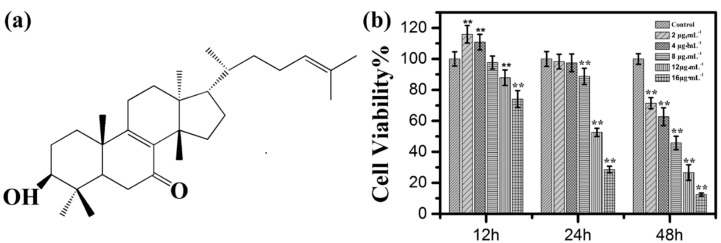
(**a**) Molecule structure of kansenone; (**b**) *In vitro* cell toxicity experiments. Relative cell viabilities of IEC-6 cells after incubation with various concentrations of kansenone for 12, 24 and 48 h, respectively. Compared with corresponding control group, ** *p* < 0.01.

Kansenone is a compound isolated from a traditional Chinese medicine plant *kansui*. To identify the acquirement of kansenone, proton nuclear magnetic resonance spectroscopy (^1^H-NMR) was employed. As shown in Figure S1 and Table S1, the ESI-MS of kansenone with a molecular ion peak at *m*/*z* 441, which corresponds to the chemical structure of kansenone in Figure 1a, and the detailed ^1^H-NMR data of kansenone that was also consistent with the previous research [11], revealed the achievement of kansenone. Kansenone was isolated by HPLC and the purity is above 98%.

In order to detect whether kansenone could suppress cell proliferation, the MTT assay was performed based on the mechanism that yellow MTT is reduced to purple formazan by cellular mitochondrial dehydrogenase in live cells [23]. Therefore, the amount of formazan produced is directly proportional to the number of living cells. IEC-6 cells were treated with increasing concentrations of kansenone, which were 2, 4, 8, 12, and 16 μg·mL^−1^ for 12, 24, and 48 h respectively. As Figure 1b shows, cell viability decreased with the increasing concentration and incubation time, indicating the inhibitory effects of kansenone on IEC-6 cells were in a dose- and time-dependent manner. The results also demonstrated that the inhibitory rate for 48 h were significantly higher than that of 12 and 24 h. The IC_50_ value of kansenone against IEC-6 cells were approximately 8.70 μg·mL^−1^ (about 19.76 μM) at 48 h. Thus, 48 h was chosen as the appropriate time to treat cancer cells in the following experiments.

**Figure 2:**
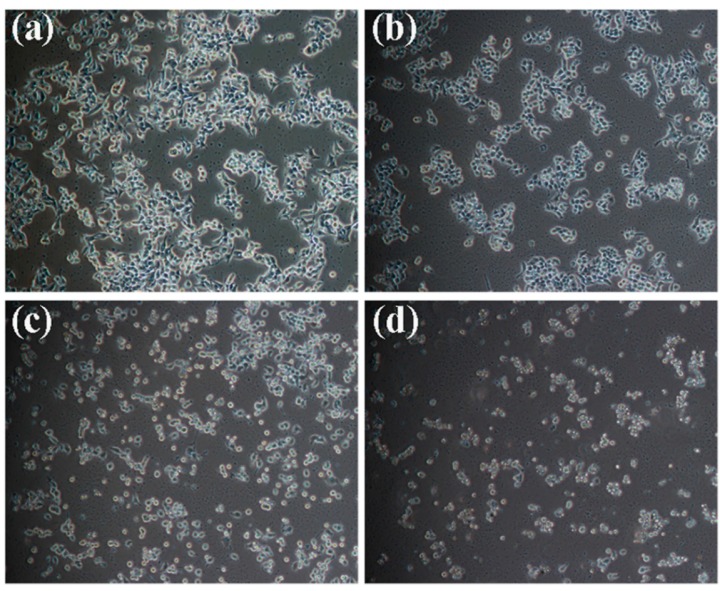
Kansenone-induced inhibitory cell proliferation in IEC-6 cells with different concentrations for 48 h, which was visualized using a microscope (magnification, ×200). (**a**) Control; (**b**) 4 μg·mL^−1^; (**c**) 8 μg·mL^−1^; (**d**) 16 μg·mL^−1^.

MTT assays indicated kansenone could effectively inhibit cell proliferation. This result was also confirmed by observing cells under bright inverted microscopy. IEC-6 cells were incubated with kansenone with the different concentrations of 4, 8, and 16 μg·mL^−1^ for 48h. After incubation with 4 μg·mL^−1^ (Figure 2b), the number of cells decreased, compared to the control group (Figure 2a). When the concentration of kansenone increased to 8 and 16 μg·mL^−1^, cell morphology became smaller in size and more round shaped, resulting in cells detaching from the surface of the Petri dish (Figure 2c, d). These photos demonstrated that kansenone caused an alteration of the cellular morphology and cell apoptosis.

### 2.2. Effects of Kansenone on Cell Cycle

Cell cycle progress involved G0 phase, G1 phase, S phase, G2 phase, and M phase. In G0 phase, cells are quiescent and stop dividing. While in G1 phase, cells increase in size and are ready for DNA synthesis replication that occurs during S phase. The G2 checkpoint is the gap between DNA synthesis and mitosis, and ensures that everything is ready to enter the M (mitosis) phase that cells orderly divide into two daughter cells [24]. In different phases of the cell cycle, the appropriate DNA repair can take place or induce irreversible senescence or apoptosis [25].

**Figure 3:**
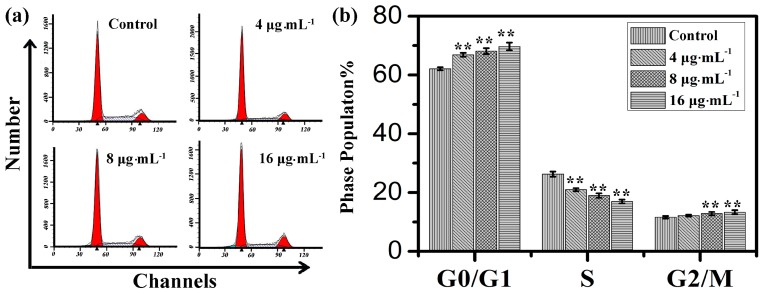
(**a**) Flow cytometry analysis on cell cycle of IEC-6 treated with kansenone of different concentration for 48 h; (**b**) Cell cycle distribution of IEC-6 cells. Compared with corresponding control group, ** *p* < 0.01.

Cell cycle distribution is generally considered as a primary parameter in cell survival, growth and proliferation. To explore the mechanism of toxicity effects on IEC-6 cells, cell cycle distribution was evaluated via flow cytometry analysis. With the increasing concentration of kansenone, the proportion of cells in G0/G1 phase increased from 62.1% to 69.7% and in G2/M phase increased from 11.6% to 13.3%, respectively, while the proportion of cells in S phase reduced from 26.3% in control cells to 17.0% after the treatment with kansenone for 48 h (Figure 3), revealing kansenone predominantly induced G0/G1 cell cycle arrest on IEC-6 cells in a dose-dependent manner.

### 2.3. Effects of Kansenone on Cell Damage

**Figure 4:**
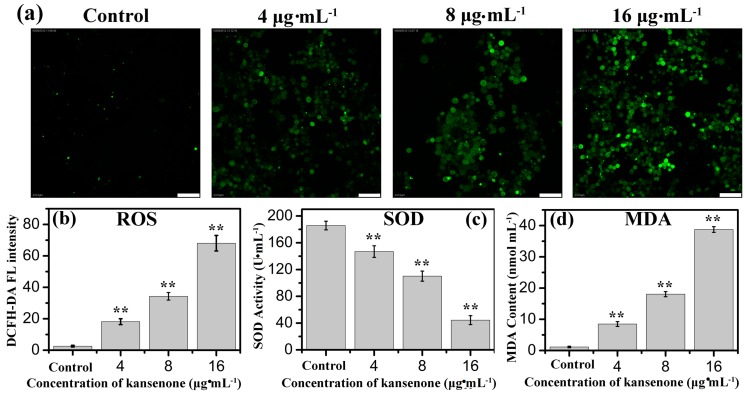
(**a**) Fluorescence images of ROS generation and effects of kansenone on cellular; (**b**) ROS; (**c**) SOD and (**d**) MDA levels. The scale bar is 50 μm. Compared with corresponding control group, ** *p* < 0.01.

ROS including superoxide anions (O_2_^−^), hydroxyl radicals, and hydrogen peroxide (H_2_O_2_), is likely to cause wide-ranging damages to proteins, nucleic acids and lipids, and lead to cell death [26]. Several intracellular antioxidant enzymes against ROS, such as SOD, catalase (CAT), and peroxidase (POD) and glutathione reductase (GR), are capable of removing oxygen radicals and their derivatives to alleviate oxidative damages and balance the physiological environment [27,28]. If the expressed level of ROS exceeds the removal capacity of antioxidant enzymes, the excessive ROS will react with polyunsaturated fatty acids to regulate cell proliferation and cell apoptosis, and in this reaction process MDA released [29]. Therefore, SOD activity and MDA content can serve as two markers for the overexpression of ROS. ROS was monitored with the non-fluorescent probe dichloroﬂuorescein diacetate (DCFH-DA) because it can change to fluorescent DCF in the presence of ROS. As shown in Figure 4a, the DCF fluorescence intensity became stronger with the increased concentration of kansenone. In Figure 4b,c, the results indicated kansenone induced a significant enhancement in ROS and reduction in SOD activity in a dose-dependent manner. The SOD activity at the highest level of kansenone (16 μg·mL^−1^) is about 44.5 U·mL^−1^ (Figure 4c), enormously lower than that of control group with 185.7 U·mL^−1^, revealing that kansenone evoked the generation of ROS. As for MDA, in contrast with SOD, MDA content increased with the increasing kansenone concentration (Figure 4d). MDA contents at 4, 8, 16 μg·mL^−1^ of kansenone are about 8.5, 10.5 and 38.7 nmol·mL^−1^, nearly 9-, 11- and 39-fold compared with the control (1.146 nmol·mL^−1^), respectively. These data revealed the ROS-mediated apoptosis mechanism of kansenone on cells. ROS overexpressed and SOD reacted with ROS. During the process where excessive ROS reacted with polyunsaturated fatty acids, large amounts of MDA were released.

### 2.4. Effects of Kansenone on Cell Apoptosis

**Figure 5:**
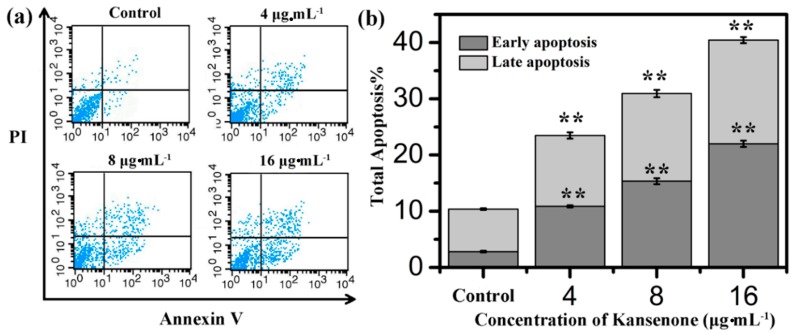
(**a**) Flow cytometry analysis on cell apoptosis of IEC-6 treated with kansenone of different concentration for 48 h; (**b**) Quantitative analysis of the number of apoptotic cells. Compared with corresponding control group, ** *p* < 0.01.

**Figure 6:**
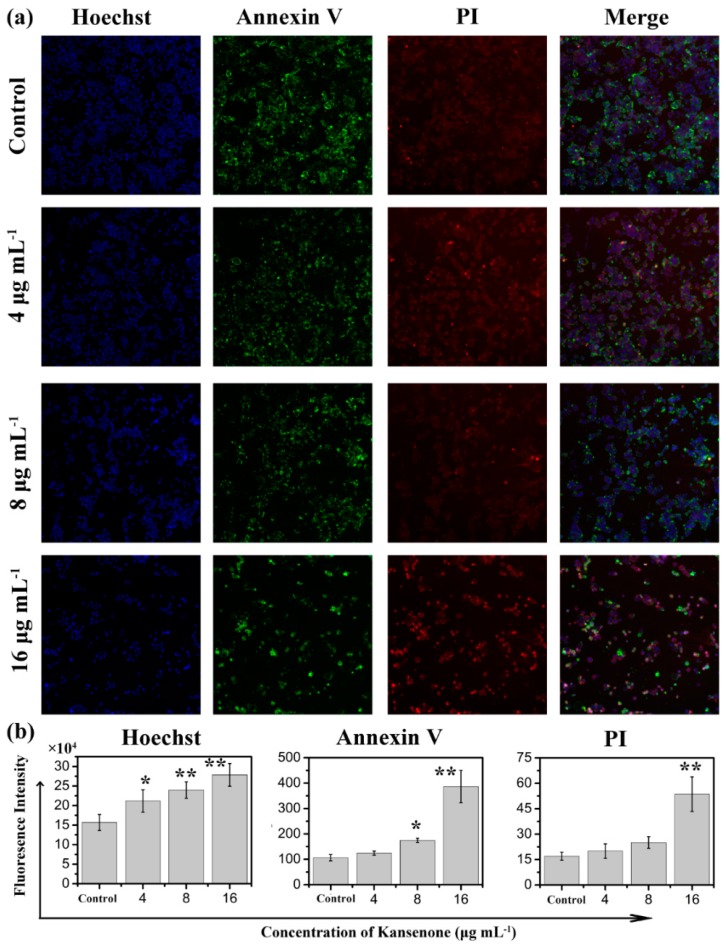
Confocal laser scanning microscopy (CLSM) images of IEC-6 cells incubated with Hoechst 33342/Annexin V-FITC/PI under a magnification of 100. (**a**) and the fluorescence intensity analysis after IEC cells incubated with Hoechst 33342/Annexin V-FITC/PI (**b**). Compared with corresponding control group, * *p* < 0.05, ** *p* < 0.01.

Programmed cell death, short for apoptosis, is related to many physiological growth control mechanisms that regulate cell proliferation and tissue homeostasis. It is a major form of cell death [30,31]. Flow cytometry was used to quantitatively analyze the apoptotic effect of kansenone *in vitro* via Annexin V-FITC/PI dual staining assay (Figure 5a). Phosphatidylserine (PS) moieties flip out from the inside of the cell membrane when apoptosis is triggered leading to specific binding of Annexin V- FITC to PS of cells in the early stage of apoptosis [32]. PI stains the necrotic cells and late apoptotic cells. As shown in Figure 5b, both the percentages of early apoptotic cells and late apoptotic cells increased with increases in kansenone concentration (4, 8 and 16 μg·mL^−1^) for IEC-6 cells. The percentages of total apoptotic cells treated with kansenone obviously changed from 23.48% to 40.43% when the concentration increased from 4 to 16 μg·mL^−1^ after 48 h treatment, whereas the proportion of apoptotic cells was only 10.37% in the control. The percentage of total apoptotic cells demonstrated that kansenone effectively promotes cell apoptosis in a concentration-dependent manner. This conclusion was further confirmed by Hoechst 33342/Annexin V-FITC/PI triple staining analysis under fluorescence microscope. In this assay, another dye Hoechst 33342 was introduced to characterize the cells nucleic. Hoechst 33342 could easily enter into apoptotic cells, but not into cells in good condition. As seen in Figure 6a, the number of cells with blue fluorescence from Hoechst 33342 gradually decreased from low to high concentrations of kansenone, indicating that cell proliferation was remarkably suppressed after kansenone treatment, consistent with the results in Figure 2. As illustrated in Figure 6b, the fluorescence intensity of Hoechst 33342, FITC and PI was significantly enhanced when IEC-6 cells were treated with kansenone at high concentrations of about 16 μg·mL^−1^, indicating that high concentration kansenone induced significant cell apoptosis, as expected in the Annexin V-FITC/PI dual staining assay.

**Figure 7:**
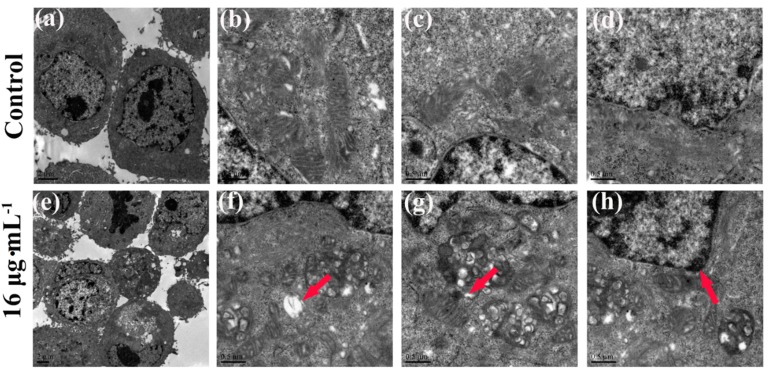
TEM images of cell ultrastructure of IEC-6 cells before (**a**–**d**) and after (**e**–**h**) treatment with kansenone of 16 µg·mL^−1^ for 48 h. Red arrow in (**f**) showed the mitochondria vacuoles. Red arrow in (**g**) showed broken mitochondria crista. Red arrow in (**h**) showed nuclear chromatin condensation.

Apoptotic signaling within the cell normally involves two fundamental pathways: the extrinsic death receptor pathway, and the intrinsic mitochondria-initiated pathway [17,33]. As Figure 7 shows, after treatment with kansenone of 16 µg·mL^−1^, IEC-6 cells were significantly damaged, compared with the control group. In Figure 7a–d, cells were arranged regularly with round shape, and well-structured mitochondria in the cytoplasm. While in Figure 7e–h, some vacuoles appeared; (e) and mitochondria were irregularly sized and arranged with mitochondria cristae break (f). Rupture was observed in some mitochondria; (g) Chromatin condensation, aggregation at the periphery of the nucleus and organelle degeneration were seen in early apoptosis cells (h). These results suggest that kansenone-induced cell apoptosis occurs via the mitochondria-initiated pathway.

**Figure 8:**
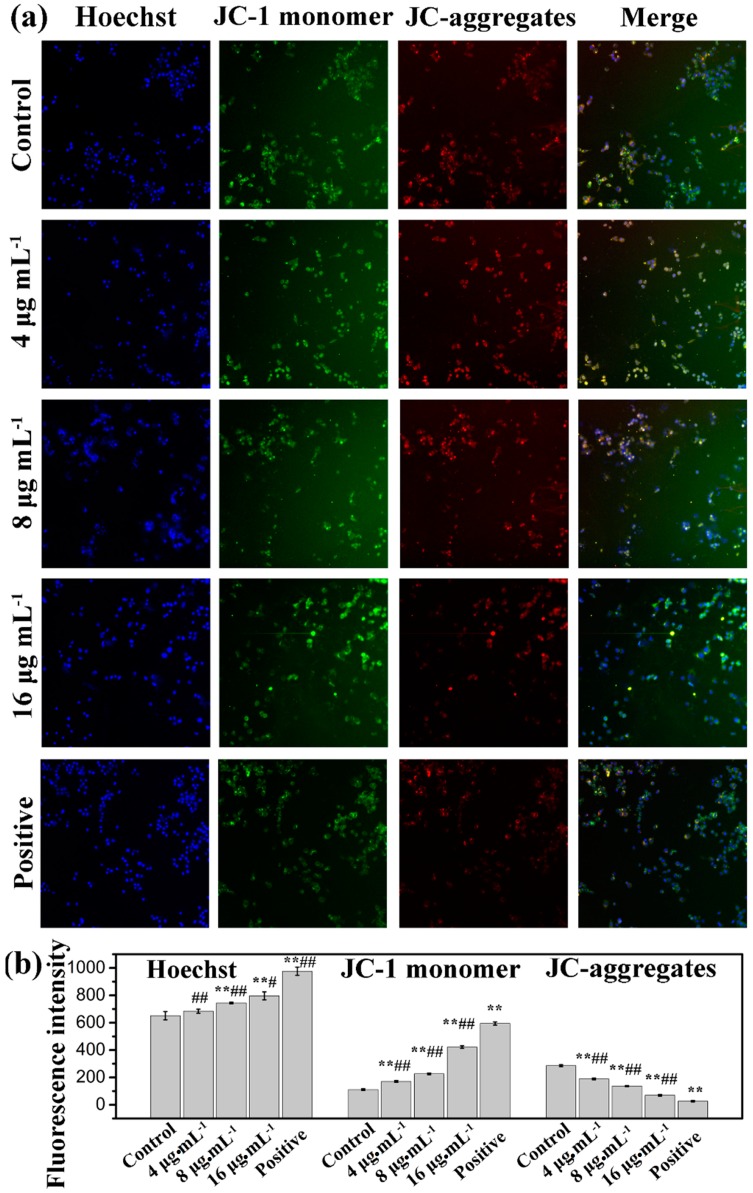
Mitochondrial membrane potential detection by high content screening (HCS) after treatment with kansenone for 48 h under a magnification of 100. Compared with corresponding control group, ** *p* < 0.01; Compared with corresponding positive control group, ^#^ *p*< 0.05, ^##^ *p* < 0.01.

The mitochondrion is a central organelle to produce cellular energy and has a vital role in programmed cell death. Therefore, we analyzed disruption or loss of the mitochondrial membrane potential (MMP) by a fluorescent dye (5,5′,6′,6-tetrachloro-1,1′,3,3′-tetraethylbenzimidazolcarbocyanine iodide, JC-1), which is capable of selectively entering mitochondria to form monomers that emit green fluorescence at low MMP, and form JC-1 aggregates that emit red fluorescence at high MMP [34,35]. Cells were treated with kansenone for 48 h and stained with JC-1 dye. As shown in Figure 8, the intensity of green fluorescence (JC-monomer) was enhanced, while red fluorescence (JC-aggregates) reduced in response to the increase of kansenone in a dose-dependent manner, which suggests that kansenone-caused depolarization of the mitochondrial membrane potential may be a core mechanism for the induction of apoptosis of IEC-6 cells that is consistent with the results in Figure 7.

**Figure 9:**
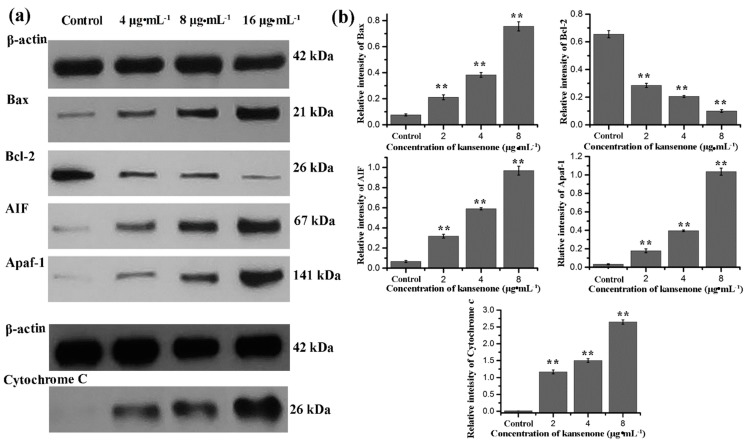
(**a**) Western blot analysis for Bax, Bcl-2, AIF, Apaf-1, and cytochrome *c* after treatment with kansenone for 48 h; (**b**) Relative intensity value of Bax, Bcl-2, AIF, Apaf-1, and cytochrome *c*. β-actin was used as an internal control. Compared with corresponding control group, ** *p* < 0.01.

**Figure 10:**
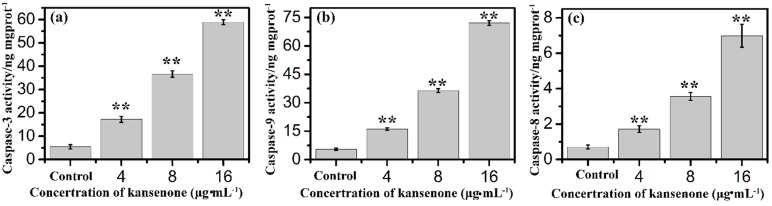
Effects of kansenone on (**a**) caspase-3; (**b**) caspase-9 and (**c**) caspase-8 activity after treatment with kansenone for 48 h. Compared with corresponding control group, ** *p* < 0.01.

To further investigate the mechanism involved in kansenone-induced mitochondrial membrane mediated apoptosis, we measured the expression of Bcl-2 family members and cytochrome *c*. The Bcl-2 family proteins consist of both the pro-apoptotic Bax and anti-apoptotic Bcl-2 proteins that regulate the mitochondrial membrane potential and caspase activation [36,37]. Cytochrome *c* can combine with Apaf-1 and procaspase-9, initiates the activation of caspase-9, which eventually activates downstream effector caspase-3 leading to the activation of the execution phase of apoptosis [38,39]. In addition, AIF usually trapped between cell membranes was released to the cytoplasm when cell apoptosis was initiated. As shown in Figure 9, kansenone treatment significantly increased the expression of the pro-apoptotic protein Bax, AIF, and Apfa-1, and down-regulated the expression of the anti-apoptotic protein Bcl-2. On the other hand, the expression of cytochrome *c* was up-regulated along with the increase of caspase-9 and caspase-3 (Figure 10a,b). These results suggested that kansenone increased the expression of Bax, AIF, Apfa-1, cytochrome *c*, caspase-3, caspase-9, and decreased Bcl-2 expression in IEC-6 cells in time- and dose-dependent manners, respectively, indicating kansenone induced cell apoptosis via the mitochondrial-mediated pathway.

**Figure 11:**
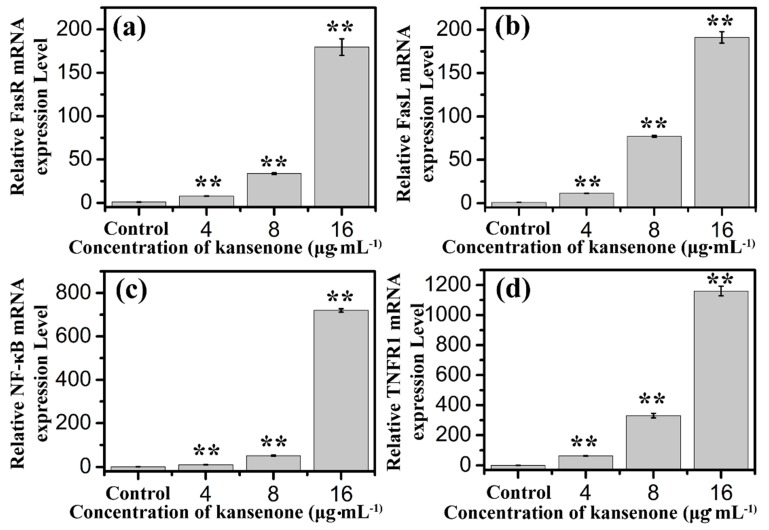
Effects of kansenone on (**a**) FasR; (**b**) FasL; (**c**) NF-κB and (**d**) TNFR1 mRNA expression level after treatment with kansenone for 48 h. Compared with corresponding control group, ** *p* < 0.01.

In addition to the intrinsic mitochondria-initiated pathway, there is another apoptotic signaling pathway, the extrinsic death receptor pathway. In this apoptotic process, drug stimulates apoptosis via death receptors, such as FasR, TNFR, DR4, DR5 [40]. FasR, known as apoptosis antigen 1 (APO-1 or APT), is encoded by tumor necrosis factor receptor superfamily member 6 (TNFRSF6) gene [41,42]. TNFR1, an integral membrane protein with an intracellular death domain, can activate caspase-8 through the adaptor protein Fas-associated death domain (FADD) outside the cell surface [43,44]. Fas and TNFR1 trigger apoptosis upon engagement by their cognate death ligands FasL and TNF [19]. NF-κB is a protein complex that controls transcription of DNA, and plays a key role in regulating the immune response to infection [45]. To ascertain whether kansenone promotes apoptosis in IEC-6 cells via a receptor-mediated pathway, caspase-8 was detected by Elisa methods and FasR, FasL, TNFR and NF-κB mRNA levels were assessed by RT-PCR. As Figure 10c and Figure 11 show, intrinsic pathway-associated genes, FasR, FasL, TNFR and NF-κB mRNA were up-regulated and cleaved caspase-8 was down-regulated in a concentration-dependent manner. These data provides additional evidence that kansenone-induced apoptosis is also mediated by the death receptor pathway.

**Figure 12:**
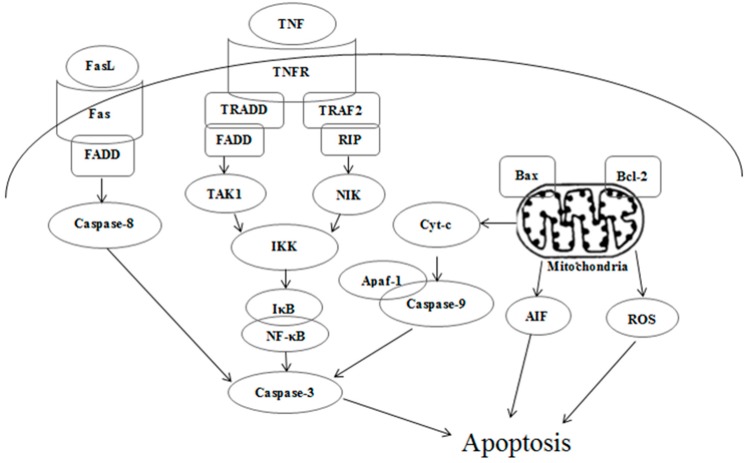
Death receptor and mitochondrial mediated apoptotic pathways induced by kansenone.

According to all these results, the toxicity mechanism of kansenone could likely be uptake and induced apoptosis of IEC-6 cells through both the death receptor and mitochondrial pathways as Figure 12 illustrates. In the death receptor pathway, receptors such as TNFR and Fas receptor interact with their corresponding ligands, which induce the formation of a death-inducing signaling complex (DISC), resulting in the release of active caspase-8 and NF-κB into the cytosol through an adaptor molecule such as FADD or TNFR1-associated death domain protein (TRADD). In the mitochondrial pathway, oxidative stress-induced cell damage initiated the release of cytochrome *c*, apoptogenic factors such as AIF, and pro-apoptotic protein such as Bax. In the presence of ATP or dATP, cytochrome c binds to Apaf-1 to form a complex that recruits and activates the initiator caspase-9. Both caspase-8 and caspase-9 could activate caspase-3, which results in cell death.

## 3. Experimental Section

### 3.1. Chemicals and Regents

Dulbecco’s Modified Eagle’s Medium (DMEM) was purchased from Gibco Co., Ltd. (Grand Island, NY, USA). Fetal bovine serum (FBS) and calf serum (CS) were purchased from Sijiqing Biological Engineering Material Co., Ltd. (Hangzhou, China). SOD and MDA kits were bought from Nanjing Jiancheng Bioengineering Institute (Nanjing, China). Propidium iodide (PI), 3-(4,5-dimethylthiazol-2-yl)-2,5-diphenyltetrazolium bromide (MTT) and RNase A were obtained from Sigma-Aldrich Co. LLC. (St. Louise, MO, USA). Mitochondrial membrane potential, ROS, Hoechst33343, and Annexin V-FITC/PI apoptosis detection kit were purchased from Beyotime Biotechnology Co., Ltd. (Nantong, China). Caspase-3, caspase-8, caspase-9 Elisa kits and all antibodies were purchased from Nanjing Saiyan Bioengineering Institute (Nanjing, China). PBS buffer was purchased from Boster Bio-engineering Co., Ltd. (Wuhan, China). Dimethylsulfoxide (DMSO) was purchased from Sinopharm Chemical Reagent Co., Ltd. (AR grade, Shanghai, China).

### 3.2. Sample Preparation

Kansenone was extracted from *kansui* as previously reported [16]. The dried and crushed roots of *kansui* were extracted with 95% ethanol under 50 °C water bath. The supernatant was separated and evaporated under reduced pressure. The retained residue was partitioned with EtOAc to provide the EtOAc fraction that was subjected to silica gel column chromatography to acquire pure kansenone, combining with preparative HPLC. Kansenone was dissolved in dimethyl sulphoxide (DMSO) at a concentration of 10 mg·mL^−1^ and diluted with DMEM medium to the indicated concentration before use.

### 3.3. Cell Culture and Cytotoxicity Assays

The rat intestinal epithelioid cell line, IEC-6 cells, were obtained from the American Type Culture Collection (ATCC) and maintained in high glucose Dulbecco’s modified Eagle’s medium (DMEM) containing 10% (*v*/*v*) fetal bovine serum, 10% (*v*/*v*) heat inactivated FCS, 1% penicillin, and 1% streptomycin at 37 °C with 5% (*v*/*v*) CO_2_ in humidified air. Cell viability was measured by MTT assay. For this procedure, IEC-6 cells were seeded in 96-well plates at a density of 1 × 10^4^ cells per well. After 24 h incubation, the medium was replaced with 100 μL of fresh medium containing kansenone at final concentrations of 0 (control), 2, 4, 8, 12, and 16 μg·mL^−1^. After 12, 24 and 48 h, respectively, cells in each well were incubated with 20 μL of MTT (5 mg·mL^−1^) at 37 °C for an additional 4 h. The supernatant was discarded and 150 μL DMSO was added into each well. The plate was gently shaken for 10 min at room temperature. The optical density (OD) was read at a wavelength of 490 nm using a Microplate Reader (Bio-Rad Model 550, Hercules, CA, USA). Each experiment was repeated at least five times. Relative cell viability was expressed as: ((OD)_test_/(OD)_control_) × 100%.

### 3.4. Cell Morphology Analysis

IEC-6 cells were seeded in 6-well plates at a density of 1.6 × 10^5^ cells per well in 1.6 mL medium solution. After 24 h incubation, the supernatant was removed and 1 mL of fresh medium containing kansenone at 4, 8, and 16 μg·mL^−1^ was added. After 48 h, cells were observed under inverted microscope. 1 mL of fresh medium without kansenone was added as a control. Each experiment was repeated three times.

### 3.5. Measurement of Intracellular Reactive Oxygen Species

To assess the intracellular ROS levels, IEC-6 cells were first plated into a 6-well plate at 1.6 × 10^5^ cells/well for 24 h, and then treated with kansenone. After 48 h incubation, cells were washed with PBS for three times and subsequently incubated with 10 μM DCFH-DA. Within the cells, DCFH-DA is converted to DCFH, which can be oxidized to the fluorescent compound DCF in the presence of ROS. After incubation for 30 min, the cells were washed twice with PBS and analyzed under confocal microscopy.

### 3.6. Measurement of SOD Activity, MDA Contents, Caspase-3, Caspase-8, and Caspase-9

The enzymatic activities of SOD, the contents of MDA, caspase-3, caspase-8, and caspase-9 were measured spectrophotometrically (Hitachi U-3010, Tokyo, Japan) using the corresponding kits from the Nanjing Jiancheng Bioengineering Institute. IEC-6 cells were seeded in 6-well plates at a density of 1.6 × 10^5^ cells per well. After 24 h incubation, 1 mL of fresh medium containing kansenone at 4, 8, and 16 μg·mL^−1^ was added. After 48 h, the supernatants were collected and detected according to the manufacturer's instructions of the kits. Absorbance of the supernatant was recorded at 550 nm for SOD, 532 nm for MDA, 450 nm for caspase-3, caspase-8, and caspase-9, respectively.

### 3.7. Cell Cycle Analysis

The cell cycle distribution of the cells was analyzed using a flow cytometer. IEC-6 cells were seeded in 6-well plates at a density of 1.6 × 10^5^ cells per well and cultured for 24 h. Then the medium was replaced with 1 mL of fresh medium containing kansenone at concentrations of 4, 8, and 16 μg·mL^−1^. After 48 h, cells were trypsinized and collected by centrifugation at 1500 rpm for 5 min. Then cells were fixed in 70% ethanol for 24 h at 4 °C and washed three times with PBS. Finally, the cell pellets were stained with RNase (1 mg·mL^−1^) and PI solution (400 μL, 100 μL·mL^−1^) for 30 min in the shade and analyzed by flow cytometry (FACScalibur, Becton Dickinson, Franklin Lakes, NJ, USA). Cells with added fresh medium without kansenone was set as control. Each experiment was repeated at least three times.

### 3.8. Cell Apoptosis Analysis

Apoptotic cell death was measured via Annexin V-FITC and PI double staining followed by flow cytometry. After IEC-6 cells were treated with kansenone at different concentrations of 0 (control), 4, 8, and 16 μg·mL^−1^ for 48 h in 6-well plates, cells were trypsinized, harvested by centrifugation at 1500 rpm for 5 min and washed with cold phosphate buffered saline (PBS) three times. Then cells were dispersed in 500 μL binding buffer. Five μL Annexin V-FITC and 5 μL PI solution were successively added to stain cells in the dark at room temperature. About 15 min later, the stained cells were detected with flow cytometry within 1 h.

To further confirm the apoptosis-induced effects of kansenone, the Hoechst/Annexin V-fluorescein isothiocyanate/PI triple staining detection kit was employed to assess cell apoptosis using HCS. Briefly, cells (1 × 10^4^ cells/well) were seeded in a 96-well plate for 24 hours and treated with kansenone at different concentrations of 0 (control), 4, 8, and 16 μg·mL^−1^ for 48 h in 100 μL medium, followed by staining with Annexin V-FITC and PI solution as described previously. Subsequently, double stained cells were fixed with 4 % paraformaldehyde for 20 min and Hoechst solution for 10 min in the dark. After staining, cells were wash with PBS and observed under high content cell image system (HCS). Each assay was replicated 6 times.

### 3.9. Measurement of the Mitochondrial Membrane Potential (ΔΨm)

Mitochondrial stability was assessed using a mitochondrial membrane potential assay kit with JC-1. IEC-6 cells of 1 × 10^4^ cells/well were cultured in 96-well plates for 24 h and then treated with kansenone of 0 (control), 4, 8, and 16 μg·mL^−1^. After 48 h treatment, the cells were incubated with 62.5 μL JC-1 fluorescent dye for 20 min in the dark at 37 °C. Then, the cells were washed slowly twice with JC-1 dyeing buffer, followed by treating Hoechst (100 μL) for 10 min. The mitochondrial membrane potential was imaged using fluorescence microscopy (Olympus, Tokyo, Japan) at 550 nm excitation and 570 nm emission for JC-1. IEC-6 cells treated with CCCP of 10 μM in kits were set as positive control.

### 3.10. RNA Isolation and Real-Time PCR

Total RNA was isolated from treated IEC-6 cells using Trizol reagent (Sigma, St. Louis, MO, USA) following the protocol provided by the manufacturer. Glyceraldehyde phosphate dehydrogenase (GAPDH) was used as the invariant control. The relative expression of RNA was calculated using 2^−ΔΔ*C*t^ method [46]. The primers used for RNA analysis were illustrated in Table 1.

**Table 1:** Primers used in real-time PCR analyses.

RNA	Sense (5ʹ→3ʹ)	Antisense (5ʹ→3ʹ)
Rat-GAPDH	TCAAGAAGGTGGTGAAGCAG	AGGTGGAAGAATGGGAGTTG
Rat-Fas	AAGATGCAGCTGAGCAGAAA	GGATTAAAGCTTGACACGCA
Rat-FasL	CACAAGGTCCAACAGGTCAG	TTCTCTTTGCCTCTGCATTG
Rat-TNFR1	CCCGTCTTCGGTCCTAGTAA	GTTGAGGGATCCGTAGAGGA
Rat-NF-κB	GTGTTCACAGACCTGGCATC	TTCAGGGTACTCCATCAGCA

### 3.11. Western Blot Analysis

Total proteins extracts were prepared from treated IEC-6 cells. The protein levels were quantified with BCA assay kit (Pierce, Waltham, MA, USA). Proteins (70 μg/well) and resolved using SDS-polyacrylamide gel, transferred to a PVDF membrane (Millipore, Burlington, MA, USA), blocked with 5% skim milk in Tris-buffered saline containing 0.1% Tween 20. Target proteins including cytochrome *c*, Bax, Bcl-2, AIF and Apaf-1 protein, were incubated with corresponding primary antibodies, and subsequently with horseradish peroxidase conjugated secondary antibodies. Protein bands were visualized using chemiluminescence (ECL) reagent (Millipore) by the Bioshine ChemitQ. After normalization by the corresponding expression of β-actin, protein expression levels were quantified using Quantity One 4.4.1 (Bio-Rad Laboratories, Hercules, CA, USA).

### 3.12. Statistical Analysis

Statistical analysis was performed by one-way analysis of variance (ANOVA) and a two-tailed Students’s with Statistica 10.0 software. All experimental values were presented as means ± SD. *p*-values < 0.05 were considered to be statistically significant.

## 4. Conclusions

In conclusion, kansenone exerts high cytotoxicity to IEC-6 cells with IC_50_ values of 8.7 μg·mL^−1^ after 48 h incubation, demonstrating the ability of kansenone to repress cell proliferation effectively. The SOD activity was down-regulated and MDA content was up-regulated with increased concentration of kansenone in a dose-dependent manner, indicating that kansenone exhibits its toxicity through the intrinsic ROS-mediated mitochondrial pathway. The proportion of cells in S phase was reduced from 26.3% to 17.0% after treatment with kansenone for 48 h, revealing kansenone predominantly triggered cell cycle arrest at the G0/G1 phase in IEC-6 cells, followed by an increase in apoptotic cell death. Annexin V-FITC/PI dual staining assay and Hoechst 33342/Annexin V-FITC/PI triple staining analysis further confirmed kansenone’s role in significant cell apoptosis. To further confirm the apoptosis pathway, TEM and JC-1 mitochondrial membrane potential, western blot and RT-PCR analyses were employed. The results demonstrated that kansenone caused mitochondrial damage, and that the mitochondrial membrane potential decreased, and up-regulated the apoptotic proteins Bax, AIF, Apaf-1, and cytochrome *c*, caspase-3, caspase-9, and down-regulated the anti-apoptotic proteins Bcl-2 related to the intrinsic pathway. Kansenone could however up-regulate caspase-8, FasR, FasL, NF-κB, and TNFR1 mRNA expression levels related to the extrinsic pathway.

## Supplementary Materials

Supplementary materials can be found at http://www.mdpi.com/1422-0067/16/08/18956/s1.
