# Supplementary material for: A Natural Triterpene Derivative from Euphorbia kansui Inhibits Cell Proliferation and Induces Apoptosis against Rat Intestinal Epithelioid Cell Line in Vitro
Source: Int J Mol Sci. 2015 Aug 12;16(8):18956–75. doi: 10.3390/ijms160818956 (PMC4581281; doi:10.3390/ijms160818956)
Supplement: Supplementary file 1 [file ijms-16-18956-s001.pdf]

Supplementary Information

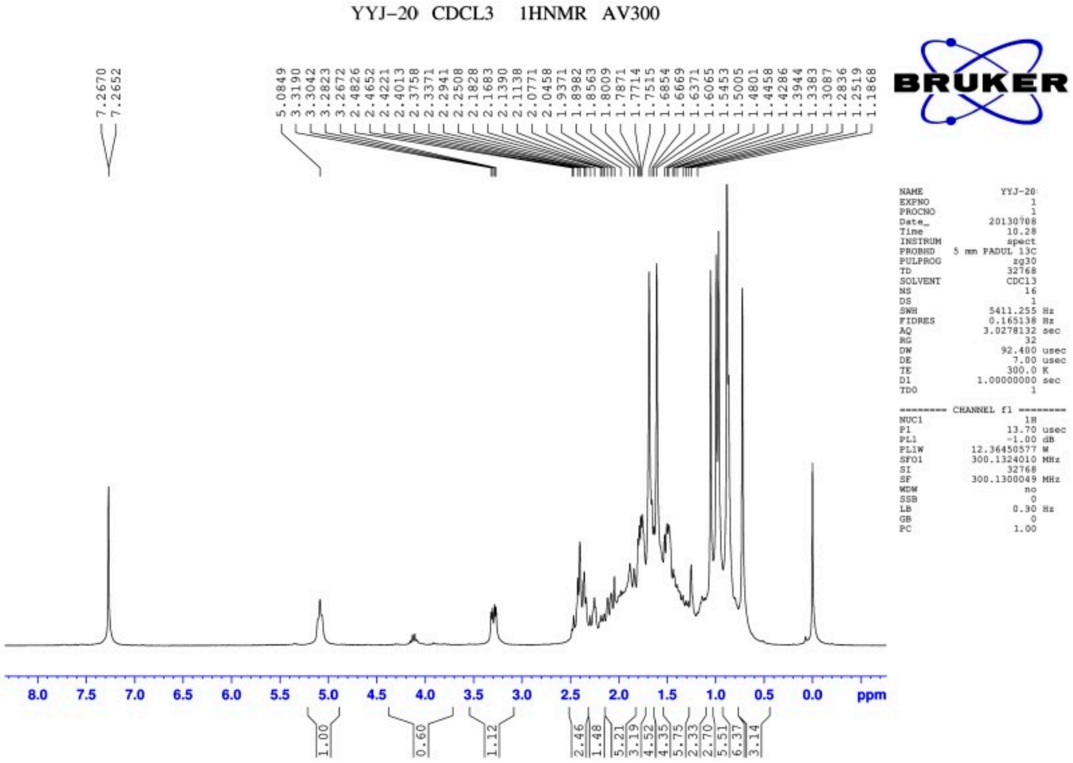

Figure S1. ^{1}H-NMR spectrum of kansenone.

Table S1. The ^{1}H-NMR data for kansenone.

Position	Kansenone in Reference [11]	Our Kansenone
1α	1.45 m	1.45 m
1β	1.86t (*J* = 13.1, 3.3 HZ)	1.86 m
2	1.75 m	1.75 m
	1.67 m	1.67 m
3α	3.29dd (*J* = 4.6, 11.6 HZ)	3.29dd (*J* = 4.6,11.6 HZ)
5α	1.67 m	1.67 m
6α	2.40dd (*J* = 3.9, 15.8 HZ)	2.40 m
6β	2.38dd (*J* = 12.4, 15.8 HZ)	2.38 m
11α	2.37 m	2.37 m
11β	2.24ddd (*J* = 4.2, 4.2, 20.4 HZ)	2.25 m
12α	1.76–1.80 m	1.75–1.80 m
15α	1.56 m	1.55 m
15β	2.13 m	2.14 m
16α	1.33 m	1.34 m
16β	1.93 m	1.94 m
17	1.43 m	1.43 m
18	0.72 s	0.73 s
19	1.05 s	1.06 s
20	1.43 m	1.43 m
21	0.88d (*J* = 6.0 HZ)	0.88d (*J* = 6.0 HZ)
22	1.13 m, 1.56 m	1.13 m, 1.55 m
23	1.90 m, 2.04 m	1.90 m, 2.05 m
24	5.08 m	5.08 m
26	1.68 s	1.69 s
27	1.61 s	1.61 s
28	0.99 s	0.99 s
29	0.88 s	0.88 s
30	0.97 s	0.97 s

Kansenone was isolated by HPLC. ^{1}H-NMR spectrum was employed to confirm the acquirement of kansenone, and the data is consistent with the reported reference.
